# Prescribing Multiple Neurostimulants during Rehabilitation for Severe Brain Injury

**DOI:** 10.1155/2014/964578

**Published:** 2014-12-22

**Authors:** Amy A. Herrold, Theresa Louise-Bender Pape, Ann Guernon, Trudy Mallinson, Eileen Collins, Neil Jordan

**Affiliations:** ^1^Edward Hines Jr. VA Hospital Research Service, P.O. Box 5000, S. Fifth Avenue (M/C 151H), Hines, IL 60141, USA; ^2^The Department of Veterans Affairs (VA), Center of Innovation for Complex Chronic Healthcare, Edward Hines Jr. VA Hospital, P.O. Box 5000, S. Fifth Avenue (M/C 151H), Hines, IL 60141, USA; ^3^Department of Psychiatry & Behavioral Sciences, Northwestern University Feinberg School of Medicine, 710 N Lake Shore Drive Chicago, IL 60611, USA; ^4^Department of Physical Medicine and Rehabilitation, Northwestern University Feinberg School of Medicine, Office of Medical Education (1574), 345 E. Superior Street Chicago, IL 60611, USA; ^5^Research Department, Marianjoy Rehabilitation Hospital, 26W171 Roosevelt Road, Wheaton, IL 60187, USA; ^6^Department of Clinical Research and Leadership, The George Washington University, 2100 Pennsylvania Avenue, Washington, DC 20037, USA; ^7^Department of Biobehavioral Health Science, College of Nursing, University of Illinois at Chicago, 845 S. Damen Avenue, Room 716, Chicago, IL 60612, USA

## Abstract

*Background. *Despite a lack of clear evidence, multiple neurostimulants are commonly provided after severe brain injury (BI). The purpose of this study is to determine if the number of neurostimulants received during rehabilitation was associated with recovery of full consciousness or improved neurobehavioral function after severe BI.* Method. *Data from 115 participants were extracted from a neurobehavioral observational study database for this exploratory, retrospective analysis. Univariate optimal data analysis was conducted to determine if the number of neurostimulants influenced classification of four outcomes: recovery of full consciousness during rehabilitation, recovery of full consciousness within one year of injury, and meaningful neurobehavioral improvement during rehabilitation defined as* either* at least a 4.7 unit (minimal detectable change) or 2.58 unit (minimal clinically important difference) gain on the Disorders of Consciousness Scale-25 (DOCS-25).* Results.* Number of neurostimulants was not significantly (*P* > 0.05) associated with recovery of full consciousness during rehabilitation, within one year of injury, or meaningful neurobehavioral improvement using the DOCS-25.* Conclusions. *Receiving multiple neurostimulants during rehabilitation may not influence recovery of full consciousness or meaningful neurobehavioral improvement. Given costs associated with additional medication, future research is needed to guide physicians about the merits of prescribing multiple neurostimulants during rehabilitation after severe BI.

## 1. Introduction

Severe brain injury (BI) results in loss of consciousness for a period of time greater than 24 hours. The transition from unconsciousness to recovery of full consciousness is described clinically according to three states of disordered consciousness: comatose, vegetative state (VS), and the minimally conscious state (MCS). Each state is defined by varying levels of arousal and awareness, ranging from the absence of arousal and sleep wake cycles in the comatose state to inconsistent but definite behavioral indicators of self- or environmental awareness in MCS [1–6].

Common practice in medical rehabilitation of people in states of disordered consciousness is to provide neuropharmacological interventions while the patient is transitioning through the states of disordered consciousness and provision is usually continued after emergence from MCS to a state of full consciousness [7]. Neurostimulants, specifically, are provided to manage arousal states which often means enhancing neural transmission [8]. The general mechanisms of action for neurostimulants are increases in the synaptic concentration of dopamine, serotonin, and noradrenaline in various brain regions [9–14]. Neurobehavioral and neurocognitive gains ascribed to neurostimulants include enhanced arousal, wakefulness, awareness, attention, memory, mental processing speed, and/or motor processing speed [7, 8].

However, neurostimulants are commonly provided to this patient population, and, until recently, there was limited evidence to support the use of neurostimulants to improve neurobehavioral function and facilitate recovery of consciousness among people in states of disordered consciousness. A randomized placebo-controlled trial published in 2012 demonstrated that a widely used neurostimulant, amantadine, is efficacious in accelerating neurobehavioral function among people in VS or MCS between 4 and 16 weeks after traumatic brain injury (TBI) [15]. Evidence for efficacy of other neurostimulants alone or in combinations such as methylphenidate is inconclusive and derived from case reports, cross-over studies of short durations where the subjects serve as their own controls (e.g., while on/off medication or over two to three days), and one meta-analysis [16]. However, off-label prescribing of neurostimulants occurs routinely [7].

Since, there is no evidence to date supporting prescription of* multiple* neurostimulants to people in states of disordered consciousness after BI for the purpose of enhancing arousal, awareness, and neurobehavioral function, we conducted exploratory analyses to provide insights about the relationship between the provision of multiple neurostimulants during rehabilitation hospitalization and recovery. The purpose of this study is to assess whether receiving* multiple* neurostimulants is associated with recovery of full consciousness or improved neurobehavioral function after severe BI.

## 2. Materials and Methods

The study sample for this paper included 115 participants selected from a larger study database of 191 people with severe brain injury (BI) who (a) were admitted to a rehabilitation hospital within 180 days of injury, (b) were ≥18 years of age at time of injury, (c) experienced unconsciousness for ≥28 consecutive days, and (d) had a severe BI that was not due to cancer, tumors, and inflammatory, infectious, and/or toxic metabolic encephalopathies. For our study sample, we included only those individuals from the larger study who had medication data and were provided at least one neurostimulant (amantadine, bromocriptine, levodopa, methylphenidate, and modafinil) during rehabilitation. Participants for the larger study were recruited from two freestanding inpatient rehabilitation facilities, one long-term acute care hospital providing rehabilitation, two Department of Veterans Affairs hospitals providing acute rehabilitation, and one subacute nursing facility. The study was approved by each facility's human subjects institutional review board. Each research participant was followed from time of rehabilitation admission through the first year of recovery to monitor time to full consciousness.

### 2.1. Data Collection and Instrumentation

Data collection procedures for the larger study included medical record reviews and bedside neurobehavioral assessments during rehabilitation hospitalization and monthly telephone follow-up to assess the recovery of full consciousness up to one year after injury. The data for the study sample were collected from 1997 to 2010.

At time of study enrollment, each participant's emergency room, intensive care, and acute care records were reviewed for sociodemographics, medical history, cause of injury, etiology, and injury-related medical conditions. After review of each subject's records, a family/surrogate interview was conducted to collect any information not obtainable from the records and/or to confirm information regarding cause of injury.

At time of rehabilitation discharge, all medications received each day of hospitalization as part of the participant's routine medical care were abstracted from the electronic medical records including start date, stop date, start time, and stop time for each dose.

#### 2.1.1. Neurobehavioral Functioning Data

During rehabilitation hospitalization, neurobehavioral evaluations using the Disorders of Consciousness Scale-25 (DOCS-25) [17–19] were conducted weekly until recovery of consciousness or completion of a sixth DOCS evaluation or discharge from the rehabilitation facility, whichever came first. The DOCS-25 is a bedside test administered by allied health clinicians [20]. Best behavioral responses, elicited with the 25 test stimuli, are scored according to a 3-point scale (0 = no response, 1 = generalized response, and 2 = localized response). The DOCS has strong interrater agreement (*κ* = .95) [17], strong reliability (alpha = .86), and strong person separation reliability (.91) all of which allow the DOCS to be used for individual patient measurement [19]. The DOCS also has excellent measurement precision, captures a broad range of function, and forms a unidimensional hierarchy with no misfitting test items and no differential item functioning across etiologies and gender [19]. There is also strong evidence of concurrent validity between the DOCS and the Glasgow Coma Scale as well as the Coma/Near-Coma Scale allowing for distinction between VS and MCS [19]. The recent [19] evidence of strong construct validity supports earlier findings [17, 19] that each DOCS test item assesses the same neurobehavioral constructs over six weeks and that the DOCS measures provide independent information about neurobehavioral functioning throughout the first six months of the recovery trajectory [17, 18]. The DOCS minimal detectable change (MDC_90_) is 4.7 indicating that this is the amount of meaningful change that exceeds measurement error. The DOCS minimal clinically important difference (MCID) is 2.58 which corresponds to the smallest amount of clinically meaningful DOCS change [21]. A final important note is that the DOCS also has strong prognostic validity for predicting recovery of consciousness for multiple time points within the first year of recovery [22] and independence with expressing needs 1 year after severe BI [23].

For the current study, we examined the total DOCS-25 measure from baseline that is collected at time of rehabilitation admission and the total DOCS-25 change measure. The total DOCS-25 change measure was computed by subtracting the baseline from the DOCS-25 measure obtained at the patient's last assessment. DOCS-25 measures were calibrated, according to rater calibrations, using the FACETS model [24] to account for patient ability, item difficulty, multiple raters, and repeated DOCS testing. The measures are reported on a 0 to 100 clinical scale [25–27].

#### 2.1.2. Full Consciousness Data

Evaluations to determine recovery of full consciousness were conducted during rehabilitation hospitalization and after rehabilitation discharge. During rehabilitation hospitalization, evaluations to determine recovery of full consciousness were conducted 1-2 times per week. After rehabilitation discharge each participant was evaluated for full consciousness one time per month up to one year after injury.

Full consciousness was defined as requiring external and internal awareness demonstrated by consistent manifestation of at least one of three criteria: (1) functional interactive communication, (2) functional use of an object, or (3) another consistent demonstration of behavior indicating awareness of the environment. The procedures for evaluating a patient to determine if they have recovered full consciousness during rehabilitation and after rehabilitation discharge are separate from and different than those of the DOCS-25.

The procedures for evaluating recovery of full consciousness are described in detail elsewhere [28]. In brief, the evaluation for recovery of full consciousness during rehabilitation is conducted by allied health clinicians via direct clinical observation and patient interactions. After discharge, the evaluation is conducted by clinicians interviewing the surrogate or caregiver according to a telephone script inclusive of probes to elicit the same behavioral data. Clinicians probe the surrogate or caregiver to collect the behavioral data that the surrogates and caregivers are knowledgeable of via direct observation and patient interactions. This behavioral information is then used with a standardized decision making algorithm where each decision point is informed by behavioral observation and patient interaction data [28].

The procedures for evaluations during rehabilitation and after discharge follow-up differ only according to the methods used to collect the same behavioral data. During rehabilitation the behavioral data is collected by clinicians via direct observation and patient interactions. During follow-up behavioral data is collected over the phone according to questions and probes made by the clinicians to the surrogate or caregiver. The DOCS-25 is not administered during this follow-up period. The behavioral information collected during the phone interview is collected via the standardized clinical probing designed to elicit the same behavioral data as collected by the surrogate and caregivers via direct observation of patient interactions. The behavioral data, collected during rehabilitation and during follow-up, are then used to inform the decision points on the algorithm used to make a determination of recovery of full consciousness.

#### 2.1.3. Medication Data

Medications received for each day of rehabilitation hospitalization as part of the participant's routine medical care were recorded according to the start/stop dates for each dose. Medication data were collected until the patient recovered full consciousness. The date of recovery of full consciousness was cross-referenced with medication data.

### 2.2. Study Sample

**Figure 1:**
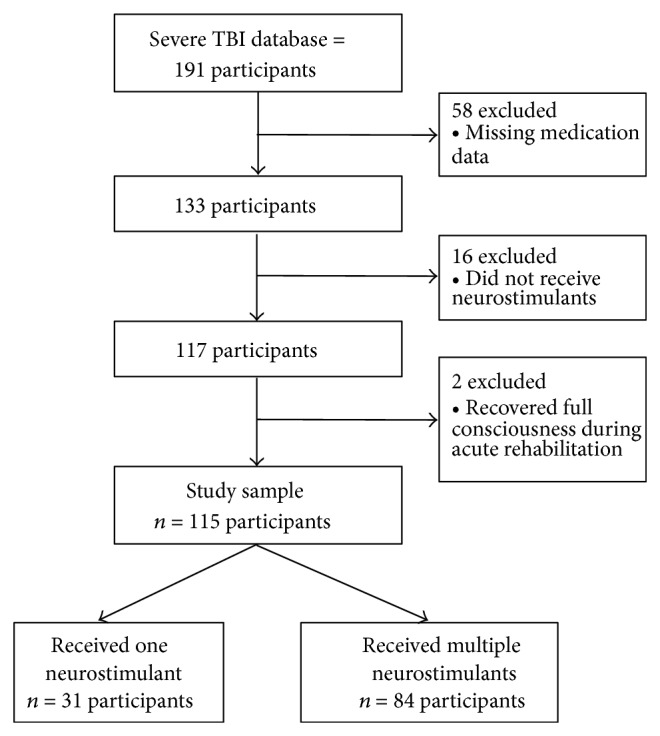
Study sample.

Of the 191 people in the larger study, medication data could not be obtained for 58 participants (i.e., missing medication data) (Figure 1). Sixteen people were not provided any neurostimulants and two people recovered full consciousness within two days of the baseline neurobehavioral assessment. After excluding these people, the final analytic sample for the current study included 115 participants. Of these 115 participants, 84 (73%) received more than one stimulant and 31 (27%) received only one neurostimulant.

### 2.3. Explanatory Variable: Number of Neurostimulants

The number of neurostimulants received during rehabilitation hospitalization was made into a binary explanatory variable: one neurostimulant or two or more neurostimulants (multiple neurostimulants). This explanatory variable was used to determine if number of neurostimulants received during rehabilitation hospitalziation was associated with the study outcomes.

### 2.4. Study Outcomes

Four outcome variables were examined and for this paper we refer to the optimal data analysis “class variable” [29] as an outcome. All outcomes are binary. The first two outcomes involved recovery of full consciousness. The first outcome was whether or not recovery of full consciousness ocurred during rehabilitation hospitalization. The second outcome was whether or not recovery of full consciousness ocurred within one year of injury. The third and fourth outcomes relate to meaningful neurobehavioral improvement. The third outcome was whether or not the participant made a gain in total DOCS-25 change measure above the DOCS-25 MDC of 4.7 [21]. The fourth outcome was whether or not the participant made a gain in total DOCS-25 change measure above the DOCS-25 MCID of 2.58 [21].

### 2.5. Data Analyses

Univariate optimal data analysis (UniODA) was conducted using UniODA software [29]. Univariate ODA is a statistical method that determines the accuracy that an explanatory variable can predict a patient's outcome classification (e.g., did or did not recover full consciousness or make meaningful neurobehavioral improvement). This method is ideal given that the purpose of the study is to examine the relationship between the number of neurostimulants received during rehabilitation and the four study outcomes. To examine significance of an explanatory variable, ODA uses Monte Carlo procedures, and leave-one-out (LOO) resampling was used to examine stability of effect strength sensitivity (ESS) [30]. ESS stability was examined because it is a normed index of likelihood of correct outcome classification and because it can be used to directly compare different UniODA models. ESS values range between 0 (classification accuracy expected by chance) and 100 (errorless classification) [29]. To compute ESS, ODA uses percentage of accuracy in classification (PAC) as follows [29]:

(1) $$\begin{array}{c} \text{PAC}=\frac{\left(\text{true}\;\text{positives}+\text{true}\;\text{negatives}\right)}{N}\times 100\%,\\ \text{Mean}\;\text{PAC}=\frac{\left(\text{Se}+\text{Sp}\right)}{C}\times 100\%,\\ \text{ESS}=\frac{\left(\text{Mean}\;\text{PAC}-50\right)}{50}\times 100\%, \end{array}$$

where *C* is number of response categories for the outcome, which for this study is two for each of the four outcomes (e.g., recovery of consciousness during rehabilitation or no recovery of consciousness during rehabilitation); Se = [true positives/(true positives + false negatives)] × 100; and Sp = [true negatives/(false positives + true negatives)] × 100.

ESS values, reflecting stability after LOO procedures, were compared for each UniODA model. An explanatory variable is considered LOO stable if ESS does not vary between the total sample and LOO analyses on the resampled total sample.

## 3. Results

### 3.1. Sample Characteristics

Table 1 summarizes the sample characteristics for the total sample, one neurostimulant, and multiple neurostimulants groups. The study sample (*n* = 115) was comprised predominantly of participants who are young (average age in years = 37 ± 16), Caucasian (75%), and males (66%). The majority of participants (57%) incurred a closed head injury. The average number of days between injury and rehabilitation was 65 ± 75. The average baseline total DOCS-25 measure was 48.46 ± 13.80 for the total sample, 50.39 ± 16.57 for the one neurostimulant group, and 47.77 ± 12.71 for the multiple neurostimulants groups. The average total DOCS-25 change measure was 2.35 ± 13.82 for the total sample, 4.12 ± 12.69 for the one stimulant group, and 1.79 ± 14.20 for the multiple neurostimulants groups. Recovery of full consciousness during rehabilitation occurred in 43% of the total sample, 43% of the one neurostimulant group, and 42% of the multiple neurostimulants group. Recovery of full consciousness within one year of injury occurred in 62% of the total sample, 57% of the one neurostimulant group, and 64% of the multiple neurostimulants group.

### 3.2. Frequencies of Neurostimulant Medications

Table 2 summarizes the frequency of neurostimulants received for the total sample, one neurostimulant, and multiple neurostimulants groups. Methylphenidate was the most common for the patients who received multiple stimulants (80%), followed by amantadine (69%) and bromocriptine (48%). Among patients who received only one neurostimulant, amantadine was most common (35%), followed closely by methylphenidate (32%).

### 3.3. Univariate Optimal Data Analyses (ODA) Results

To determine whether receiving multiple neurostimulants was associated with recovery outcomes, a separate UniODA model was completed for each outcome utilizing number of neurostimulants (i.e., one or multiple neurostimulants) as the explanatory variable in each UniODA model. Number of neurostimulants received was not significantly (*P* > 0.05) associated with recovery of full consciousness during rehabilitation, recovery of full consciousness within one year, or meaningful neurobehavioral improvement defined by the DOCS-25 MDC_90_ and MCID (Table 3).

## 4. Discussion

This unique data set collected during rehabilitation after severe BI allowed for the exploratory analyses of neurostimulants. In this sample, recovery of full consciousness during and after rehabilitation up to one year after injury and meaningful improvement in neurobehavioral function during rehabilitation were not associated with the number of neurostimulants received during rehabilitation.

Though a recent randomized, placebo-controlled trial has shown that amantadine is efficacious for facilitating neurobehavioral recovery after severe BI [15], no studies have been reported indicating that prescribing multiple neurostimulants is effective. Findings from our exploratory analyses suggest, however, that not only did the majority of the sample receive multiple neurostimulants (73%) but also recovery of full consciousness and meaningful neurobehavioral improvement are not different for those who received single or multiple neurostimulants.

The majority of our study sample was provided multiple neurostimulants and yet our findings indicate no association between use of multiple neurostimulants and improved outcomes according to four indices of recovery. Given the common off-label use of neurostimulants [7, 31] and the dearth of knowledge regarding multiple neurostimulants prescribing practices, future research is warranted in this area. One direction towards filling this knowledge gap would be a data repository collecting off-label neurostimulant medication data and rehabilitation outcome data among people in states of disordered consciousness worldwide [31] or adding the collection of neurostimulant medication data to existing nationwide data repositories. Another randomized clinical trial, or perhaps a naturalistic open-label clinical trial, may be warranted to determine if multiple neurostimulants are more efficacious than a single neurostimulant. The clinical trial could examine the same outcomes examined here or other outcomes related to a specific function. Methylphenidate, for example, may be indicated to enhance processing speed [7], whereas bromocriptine and levodopa may be indicated for movement initiation and akinesia [32]. In other words, one possible explanation for off-label multiple neurostimulants treatment, despite lack of evidence, is that different neurostimulants may be used to enhance specific aspects of neurorehabilitation. It is also plausible that multiple neurostimulants were provided one or two times to examine short term clinical effects to determine which medication if any helped with a specific function (e.g., arousability, visual tracking). Future analyses could address this by examining specific function or cognitive outcomes in order to determine if the results obtained would change if the sample was culled to reflect only participants who received multiple neurostimulants at the same time.

When there is a paucity of evidence on a common clinical approach, it is of value to conduct exploratory analyses to examine factors that may guide future research and inform clinical practice. As such, the reported findings are novel and informative but should be interpreted in light of study limitations. First, a larger data set and/or a data set with other neurobehavioral outcomes (e.g., auditory attention) and neurophysiological outcomes (e.g., brain stem auditory evoked potentials), for example, would allow elucidation of the relationship between receiving multiple neurostimulants and recovery. Among these neurobehavioral outcomes, it may be of value to also include additional measures of neurobehavioral function in future studies such as those examined in a systematic review in 2010 [33] including the Coma Near-Coma scale [34], Coma Recovery Scale-Revised [35], and Disability Rating Scale [36] as well as to compute the MDC and MCID for these measures. Another limitation is the lack of side effect data for this sample. Data on side effects associated with the use of neurostimulants in this population and side effect profiles could influence which and how many neurostimulants should be used during rehabilitation.

Future analyses should also consider the cost of these neurostimulants, as it may be more cost-effective to use only one neurostimulant rather than more than one if outcomes are not significantly different [37]. Furthermore, the addition of qualitative data regarding physicians prescribing practices may shed light on why certain patients received multiple neurostimulants and others did not. This type of qualitative data assessment on clinical decision making regarding neurostimulant prescribing practices could be conducted in future research studies.

## 5. Conclusion

Study findings suggest that receiving multiple neurostimulants during rehabilitation hospitalization does not influence recovery of full consciousness or meaningful neurobehavioral improvement. Future research is needed to determine whether it is advisable for clinicians to prescribe multiple neurostimulants during rehabilitation. Given positive findings that amantadine facilitates neurobehavioral function [15], it may be a better alternative to prescribe a single neurostimulant consistently such as amantadine.

## Figures and Tables

**Table 1:** Summary of sample characteristics.

	Total sample (*n* = 115)	One neurostimulant (*n* = 31)	Multiple neurostimulants (*n* = 84)
Age (years, mean ± stdev)	36.7 ± 16.3	35.4 ± 12.9	37.2 ± 17.5

Gender	Male: 66% (76/115)	Male: 81% (25/31)	Male: 61% (51/84)
	Female: 34% (39/115)	Female: 19% (6/31)	Female: 39% (33/84)

Ethnicity			
(i) Caucasian	75% (86/115)	81% (25/31)	73% (61/84)
(ii) African American	10% (12/115)	6% (2/31)	12% (10/84)
(iii) Asian	3% (4/115)	6% (2/31)	2% (2/84)
(iv) Hispanic	6% (7/115)	0% (0/31)	8% (7/84)
(v) Arabic	2% (2/115)	3% (1/31)	1% (1/84)
(vi) Israeli	1% (1/115)	0% (0/31)	1% (1/84)
(vii) Serbian	1% (1/115)	3% (1/31)	0% (0/84)
(viii) Unknown	2% (2/115)	0% (0/31)	2% (2/84)

Etiology			
(i) Closed head injury	57% (66/115)	55% (17/31)	59% (49/84)
(ii) Open head injury	17% (4/115)	9% (3/31)	1% (1/84)
(iii) Anoxic	20% (23/115)	16% (5/31)	21% (18/84)
(iv) Hemorrhagic	5% (6/115)	6% (2/31)	5% (4/84)
(v) Aneurysm	3% (3/115)	3% (1/31)	2% (2/84)
(vi) Blast trauma	3% (3/115)	3% (1/31)	2% (2/84)
(vii) Other	9% (10/115)	6% (2/31)	10% (8/84)

Days between injury and rehabilitation (days, mean ± stdev)	65 ± 75	71 ± 47	63 ± 83

Percentages represent the valid percent, for which the denominator is the total sample minus the missing participant cases for each specific variable.

**Table 2:** Neurostimulant medications prescribed during rehabilitation.

Medication	Total sample (*n* = 115)	One neurostimulant (*n* = 31)	Multiple neurostimulants (*n* = 84)
Amantadine	60% (69/115)	35% (11/31)	69% (58/84)
Bromocriptine	41% (47/115)	23% (7/31)	48% (40/84)
Levodopa	2% (2/115)	0% (0/31)	2% (2/84)
Methylphenidate	67% (77/115)	32% (10/31)	80% (67/84)
Modafinil	30% (34/115)	10% (3/31)	37% (31/84)

**Table 3:** Univariate optimal data analysis (UniODA) results, neurostimulants as explanatory variable.

Outcome variable	UniODA model	*N*	% satisfied	*P*	ESS
Recovery of full consciousness during rehabilitation	If received one neurostimulant, then predict recovery of consciousness during rehabilitation	30	43.3	1.000	0.8
	If received multiple neurostimulants, then predict no recovery of consciousness during rehabilitation	66	42.4	1.000^†^	−30.9^†^

Recovery of full consciousness within one year	If received one neurostimulant, then predict no recovery of consciousness within one year	30	56.7	0.506	6.8
	If received multiple neurostimulants, then predict recovery of consciousness within one year	70	64.3		

Change in total DOCS-25 score above MDC_90_ of 4.7	If received one neurostimulant, predict clinically detectable change in DOCS-25 score	22	40.9	0.800	4.1
	If received multiple neurostimulants, predict clinically nondetectable change in DOCS-25 score	70	35.7		

Change in total DOCS-25 score above MCID of 2.58	If received one neurostimulant, predict clinically detectable change in DOCS-25 score	22	50.0	0.463	7.4
	If received multiple neurostimulants, predict clinically nondetectable change in DOCS-25 score	70	40.0		

*N* indicates number of observations in a given predicted class category. % satisfied indicates percentage of observations in a given predicted class category. † indicates LOO *P* and ESS values reported due to instability. That is, if LOO ESS is lower than training ESS, then the results are not LOO stable.
